# Cone-Beam CT Assessment of the Canalis Sinuosus in an Indian Population: A Retrospective Imaging Study

**DOI:** 10.12688/f1000research.176689.2

**Published:** 2026-03-18

**Authors:** Ceena Denny, Srikant Natarajan, Hannah Haneef, Shubha BS, Divyam Sen, Aishwarya Sukumaran Nair

**Affiliations:** 1 Department of Oral Medicine and Radiology, Manipal College of Dental Sciences Mangalore, Manipal Academy of Higher Education, Manipal, India; 2 Department of Oral Pathology and Microbiology, Manipal College of Dental Sciences Mangalore, Manipal Academy of Higher Education, Manipal, India

**Keywords:** Anterior Maxilla; Canalis Sinuosus; Cone-Beam Computed Tomography; Neurovascular Anatomy

## Abstract

**Background:**

Data on Canalis Sinuosus (CS) morphology in South Asian populations remain limited, despite frequent anterior maxillary implant placements. This study aimed to evaluate the occurrence, positional characteristics, and morphometric features of the CS in Indian adults using cone-beam computed tomography (CBCT) and to assess sex-related differences.

**Methods:**

A cross-sectional analysis was conducted on 245 CBCT scans with intact maxillary incisors and canines. Multiplanar reconstructions were used to identify the CS and document its laterality, tooth relationship, and orientation. Measurements included canal diameter at the alveolar crest and distances to the alveolar crest, buccal cortical plate, and nasal floor. Inter-observer agreement was assessed using Cohen’s κ. Sex differences were analyzed using χ ^2^ and independent-samples t-tests (α = 0.05).

**Results:**

CS was detected in 67.3% of individuals, with bilateral presentation in 55.9% and unilateral in 11.4%. Detection rates were similar between sides (left: 62.4%, right: 60.8%). The canal was most frequently adjacent to the lateral incisor (44.7% left, 50.3% right), and approximately half of the canals showed palatal orientation. The mean canal diameter was 0.8 mm. Mean distances to the alveolar crest, buccal cortical plate, and nasal floor were 9.2 mm, 7.0 mm, and 11.5 mm, respectively. Males showed significantly larger canal diameters on the left side (p = 0.008), greater buccal cortical distances bilaterally (p < 0.001), and larger right nasal floor distances (p = 0.011).

**Conclusions:**

The CS is a frequently observed, typically bilateral anatomical structure in the anterior maxilla of Indian adults, mostly located palatal adjacent the lateral incisor. The morphometric and sex-specific data obtained provide valuable reference parameters for implant planning and minimizing surgical complications.

## Introduction

The canalis sinuosus, first documented by Frederic Wood Jones in 1939, is a bony canal approximately 2 mm in diameter that facilitates the passage of the anterior superior alveolar nerve (ASAN). This nerve branches from the infraorbital canal and traverses the boundaries of the maxillary sinus and nasal cavity, ultimately reaching the anterior maxilla. ^1, 2^ The ASAN extends laterally along the orbital floor, adjacent to the infraorbital nerve, and continues along the lateral wall of the nasal cavity. It then proceeds beneath the nasal floor, sometimes giving rise to an accessory branch that travels through a canal known as the Canalis Sinuosus. ^3^ Understanding the anatomical variations in the maxilla is crucial, as this area is frequently involved in surgical procedures such as the extraction of impacted teeth, dental implant placement, orthognathic surgery, and endodontic treatments. ^4^

Identifying CS on two-dimensional radiographs presents a challenge due to its potential to resemble a well-defined periapical lesion, which may result in misdiagnosis and unwarranted endodontic procedures. Additionally, its trajectory might be erroneously interpreted as a fracture. ^5^ Conversely, CBCT offers comprehensive visualization, facilitating precise detection of the CS, including its presence or absence, anatomical positioning, relationship to adjacent structures, diameter, and linear measurements, all without superimpositions. ^5, 6^

Although several CBCT-based studies have described the CS in different populations, robust data remain scarce for Indian and broader South Asian cohorts. Existing work has also paid limited attention to sex-related morphometric variation and to quantifying distances between the CS and surgically relevant landmarks in the anterior maxilla. These gaps constrain the development of population-specific guidelines and safety margins for implant placement and other anterior maxillary procedures.

Injury to the CS can lead to complications such as hemorrhage, implant failure, pain, and paraesthesia. ^7^ Therefore, this retrospective cross-sectional CBCT study aimed to (1) determine the prevalence and laterality of the canalis sinuosus in an Indian adult population, (2) characterize its tooth-related location and spatial orientation, (3) quantify its linear distances from the alveolar crest, buccal cortical plate, and nasal floor, and (4) assess potential sex-related differences in these morphometric parameters.

## Methods

A Cross-sectional retrospective study was conducted after obtaining approval by the Manipal College of Dental Sciences Institutional Ethics Committee with approval number (protocol number 24150/2025) dated 21/1/2025. The study was conducted between February 2025 and July 2025. All procedures performed involving human participants were in accordance with the ethical standards of the institutional committee and with the 1964 Declaration of Helsinki and its later amendments. Written informed consent was obtained from all participants for their involvement in the study. A total of 501 CBCT scans were screened, and those fulfilling the predefined inclusion and exclusion criteria were included in the study. Among these, 245 CBCT images were retrieved from the archives of the Department of Oral Medicine and Radiology. Purposive sampling was utilized for the selection of the sample.

All participants provided written informed consent for participation and publication of anonymized clinical data and images.

### Sample size calculation

Using the statistical parameters obtained from the study by Samunahmetoglu et al. ^5^ the sample size was calculated as 245, using the formula where:

$$n=\frac{z_{\alpha /2}^{2}p\left(1-p\right)}{e^{2}}$$

*z*_*α*/2_: the critical value from the standard normal distribution corresponding to a significance level of *α*/2

*p*: the estimated proportion of the population

*e*: the desired margin of error.

where *p* was derived from the prevalence reported in a previous CBCT study of the CS and *e* represented the desired margin of error.

**Image Acquisition**: The scans were acquired using Promax 3D Mid (Planmeca Oy, Helsinki, Finland) CBCT unit, with subsequent analysis conducted using the manufacturer’s software, Romexis 4.0.12 R (Planmeca Oy, Helsinki, Finland). The exposure parameters adhered to the standard default settings of the device, which are contingent upon the Field of View (FOV). Specifically, these parameters were set at 90 kVp, 8 mA, 12.5 s. The images were reconstructed using Romexis software version 4.6.2.R.

Medium FOV scan volumes that covered the entire maxilla with voxel size ≦200 μm were selected for the study. Patients above 16 years and without any pathologies in the anterior maxilla up to the molars were included in the study. Conversely, patients who were less than 16 years old, had undergone orthodontic treatment or orthognathic surgery were excluded. Additionally, individuals with cranial anomalies, syndromes, trauma or surgery, or endocrine disorders were also excluded. The presence of impacted teeth retained roots, dental implants, restorations, foreign bodies, or pathological lesions in the anterior maxillary area further disqualified patients from participation. Moreover, low dose CBCT images and images with artifacts were excluded to ensure image quality and accuracy.

All CBCT images were examined in axial, coronal, and sagittal planes for the presence of CS. The distance at which they emerge in relation to the nasal cavity floor, the ridge’s buccal cortical bone and the alveolar ridge crest was measured using CBCT.

**Figure 1.**
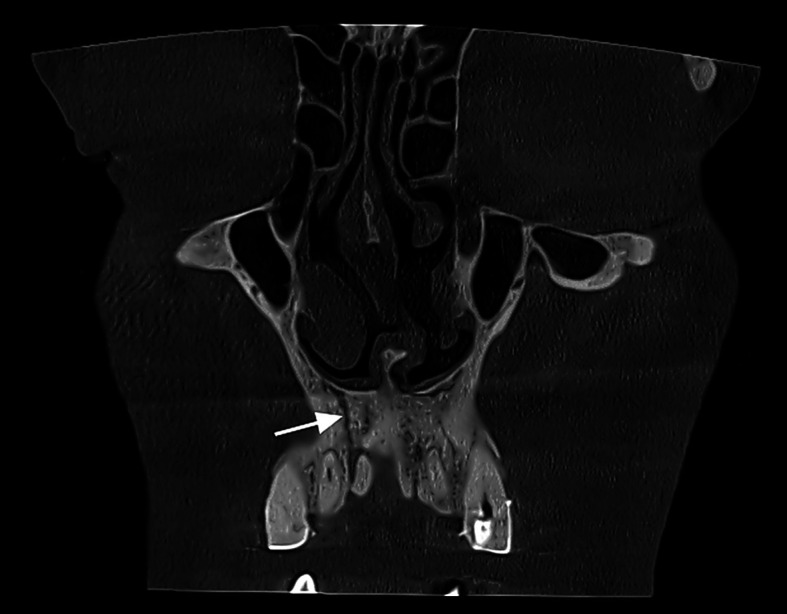
Presence of Canalis Sinuosus.

**Image Evaluation**: All the scans were evaluated for the following parameters:

Presence or absence of CS ( Figure 1)

**Figure 2.**
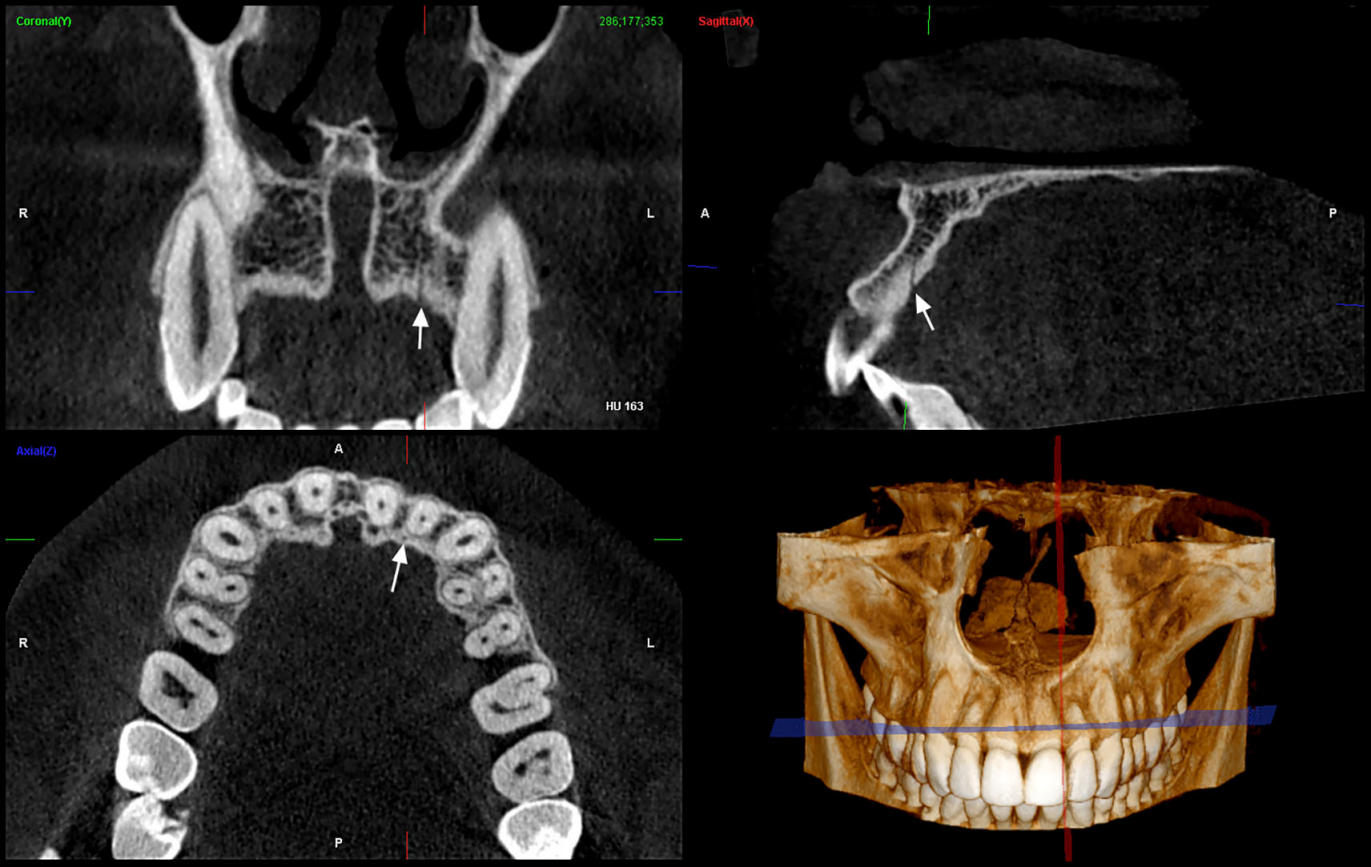
Image shows CS adjacent to the palatine root of the left central incisor in the a) Coronal, b) Sagittal, c) Axial images, d) 3D.

CS position with respect to lateral incisors in different sections ( Figure 2)

**Figure 3.**
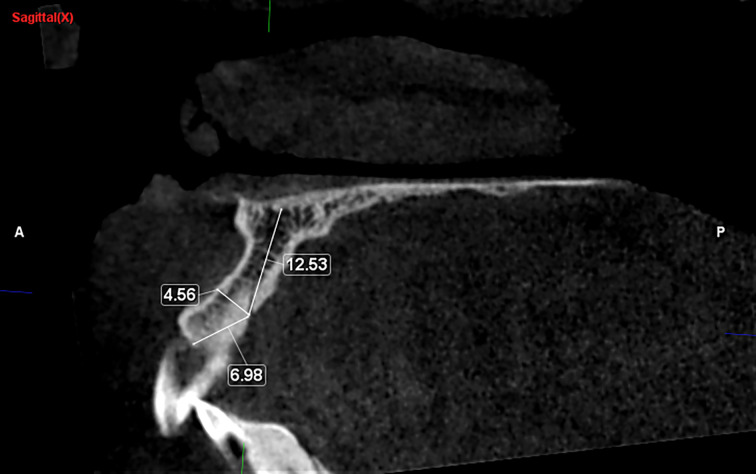
Measurement of the distance from the opening of the CS to three anatomical landmarks- the alveolar ridge crest, to the buccal cortical margin and to the floor of the nasal cavity in the cross-sectional image.

Distance of CS from cortical plates, alveolar crest and nasal floor ( Figure 3)

**Figure 4.**
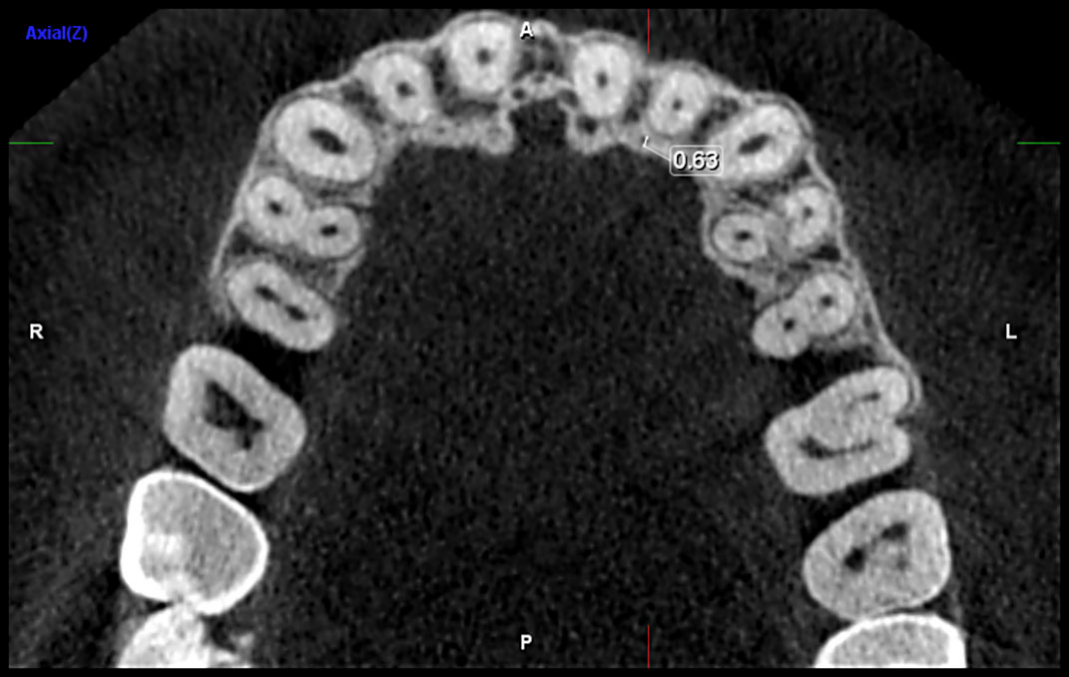
Measurement of the diameter of the CS located near the palate of the left central incisor in axial section.

Diameter of the canal ( Figure 4)

Statistical analyses were conducted utilizing the Statistical Package for Social Sciences (SPSS 25), SPSS Inc, Chicago, IL. Descriptive statistics for continuous variables were presented using mean, standard deviation, median, and interquartile range, while frequency distribution was employed for categorical variables. The Shapiro–Wilk test was used to assess normality. The canal dimensions, specifically length and diameter, were compared between sexes using an independent t-test. The presence or absence of canals was assessed using a chi-square test to compare males and females. Additionally, the proximity of the canals to the tooth was evaluated using chi-square test. A p-value of <0.05 was considered statistically significant.

## Results

### Sample characteristics

A total of 245 individuals were included in the study, with a mean age of 44.46 ± 15.15 years (median: 45). Among them, 134 (54.7%) were females and 111 (45.3%) were males.

### Detection of the CS

CS was observed in the study population in 67.3% of the patients, with 137 individuals (55.9%) exhibiting it on both sides and 28 individuals (11.4%) presenting with unilateral presence. The canal was absent in 80 individuals (32.7%). When assessed separately for each side, the CS was observed on the left in 153 individuals (62.4%) and on the right of 149 individuals (60.8%). Presence and absence of CS on left and right was evaluated by 2 observers and detection was tested for agreement using kappa statistics. Inter-observer agreement for the detection of canalis sinuses was assessed using Kappa statistics and showed good to excellent agreement (κ = 0.80), confirming the consistency of observations between the two evaluators.

### Location of CS

The analysis of the CS’s proximity to specific teeth revealed distinct patterns. On the left side, the canal was predominantly associated with the lateral incisor, accounting for 68 cases (44.7%), followed by the central incisor with 63 cases (41.4%), and the canine with 21 cases (13.8%). Conversely, on the right side, the lateral incisor showed the highest association with 76 cases (50.3%), followed by the central incisor at 50 cases (33.1%), and the canine at 25 cases (16.6%). Further examination of the spatial relationship between the CS and adjacent teeth indicated that, on the left side, the canal was positioned palatally in 81 cases (53.3%), disto-palatal in 49 cases (32.2%), and mesio-palatal in 22 cases (14.5%). A similar distribution was observed on the right side, with the canal located palatally in 75 cases (49.7%), disto-palatal in 40 cases (26.5%), and mesio-palatal in 36 cases (23.8%). Statistical analysis using Pearson’s Chi-square test confirmed a significant association between the CS location and tooth proximity on both the left (χ ^2^ = 18.164, df = 4, p = 0.001) and right sides (χ ^2^ = 9.521, df = 4, p = 0.049).

### Metric evaluation

The mean diameter of the CS near the alveolar crest was measured at 0.76 mm (±0.27) on the left side and 0.80 mm (±0.27) on the right side. The distance from the CS to the alveolar crest was recorded as 9.23 mm (±3.58) on the left side and 9.22 mm (±3.39) on the right side. The distance from the CS to the buccal cortical plate was 7.19 cm (±1.65), with a median of 7.18 cm, on the left side and 6.82 cm (±1.74) on the right side. Additionally, the distance from the CS to the floor of the nasal cavity was 11.4 cm (±3.78) on the left side and 11.6 cm (±3.64) on the right side ( Table 1).

**Table 1.** Proximity of the canalis sinuosus to adjacent teeth.

Location to tooth (Left)	Central incisor n (%)	Lateral incisor n (%)	Canine n (%)	Total	Chi square	P value
Left side						
Disto-palatal	31 (49.2)	15 (22.1)	3 (14.3)	49	18.164	0.001
Mesio-palatal	3 (4.8)	14 (20.6)	5 (23.8)	22		
Palatal	29 (46.0)	39 (57.4)	13 (61.9)	81		
Total	63 (100)	68 (100)	21 (100)	152		
Right side						
Disto-palatal	17 (34.0)	18 (23.7)	5 (20.0)	40	9.521	0.049
Mesio-palatal	5 (10.0)	25 (32.9)	6 (24.0)	36		
Palatal	28 (56.0)	33 (43.4)	14 (56.0)	75		

### Gender-based evaluation

The presence of the CS did not demonstrate a statistically significant association with sex (χ ^2^ = 3.247, p = 0.197). It was absent in 35.1% of females and 29.7% of males, present unilaterally in 8.2% of females and 15.3% of males, and present bilaterally in 56.7% of females and 55% of males. The presence of the CS on the left side revealed no significant sex-based difference, being absent in 38.1% of females and 36.9% of males, and present in 61.9% of females and 63.1% of males. Similarly, on the right side, there was no significant difference between sexes in its presence, with absence rates of 40.3% in females and 37.8% in males, and presence in 59.7% of females and 62.2% of males.

The analysis of tooth proximity on the left side revealed no significant association with sex (χ ^2^ = 0.225, p = 0.894). The highest frequency of proximity was observed near the lateral incisor (45.8% in females, 43.5% in males), followed by the central incisor (39.8% in females, 43.5% in males), and the lowest frequency was near the canine (14.5% in females, 13% in males). Similarly, on the right side, no significant difference was detected (χ ^2^ = 0.658, p = 0.720). The lateral incisor exhibited the highest frequency of proximity (47.6% in females, 53.6% in males), followed by the central incisor (34.1% in females, 31.9% in males), and the canine (18.3% in females, 14.5% in males).

The spatial orientation of the CS in relation to the tooth exhibited no statistically significant differences based on sex, on either the left side (χ ^2^ = 0.554, p = 0.758) or the right side (χ ^2^ = 3.057, p = 0.217). On the left side, the canal was predominantly located palatally (50.6% in females, 56.5% in males), followed by a disto-palatal position (33.7% in females, 30.4% in males) and a mesio-palatal position (15.7% in females, 13% in males). Similarly, on the right side, the palatal location was most prevalent (53.7% in females, 44.9% in males), succeeded by the disto-palatal (28% in females, 24.6% in males) and mesio-palatal (18.3% in females, 30.4% in males) positions ( Table 2).

**Table 2.** Canalis Sinuosus; Location and its proximity to teeth and sex-based variation.

	Categories	N	Sex		Chi square	P value
			F (N (%))	M (N (%))		
Presence of Canalis Sinuosus	Absent	80	47 (35.1)	33 (29.7)	3.247	0.197
	One side	28	11 (8.2)	17 (15.3)		
	Both sides	137	76 (56.7)	61 (55)		
Presence in Left side	Absent	92	51 (38.1)	41 (36.9)	0.033	0.857
	Present	153	83 (61.9)	70 (63.1)		
Presence in Right side	Absent	96	54 (40.3)	42 (37.8)	0.154	0.695
	Present	149	80 (59.7)	69 (62.2)		
Left Position	0	92	50 (37.6)	42 (37.8)	3.632	0.934
	1	29	13 (9.8)	16 (14.4)		
	2	39	21 (15.8)	18 (16.2)		
	3	13	8 (6)	5 (4.5)		
	4	31	19 (14.3)	12 (10.8)		
	5	15	8 (6)	7 (6.3)		
	6	3	1 (0.8)	2 (1.8)		
	7	3	1 (0.8)	2 (1.8)		
	8	14	9 (6.8)	5 (4.5)		
	9	5	3 (2.3)	2 (1.8)		
Right Position	0	94	52 (38.8)	42 (37.8)	8.486	0.486
	1	28	14 (10.4)	14 (12.6)		
	2	33	21 (15.7)	12 (10.8)		
	3	14	9 (6.7)	5 (4.5)		
	4	17	12 (9)	5 (4.5)		
	5	18	9 (6.7)	9 (8.1)		
	6	5	2 (1.5)	3 (2.7)		
	7	5	2 (1.5)	3 (2.7)		
	8	25	9 (6.7)	16 (14.4)		
	9	6	4 (3)	2 (1.8)		
Tooth Proximity Left	Central Incisor	63	33 (39.8)	30 (43.5)	0.225	0.894
	Lateral Incisor	68	38 (45.8)	30 (43.5)		
	Canine	21	12 (14.5)	9 (13)		
Tooth Proximity Right	Central Incisor	50	28 (34.1)	22 (31.9)	0.658	0.72
	Lateral Incisor	76	39 (47.6)	37 (53.6)		
	Canine	25	15 (18.3)	10 (14.5)		
Location to tooth Left	Disto Palatal	49	28 (33.7)	21 (30.4)	0.554	0.758
	Mesio Palatal	22	13 (15.7)	9 (13)		
	Palatal	81	42 (50.6)	39 (56.5)		
Location To tooth Right	Disto Palatal	40	23 (28)	17 (24.6)	3.057	0.217
	Mesio Palatal	36	15 (18.3)	21 (30.4)		
	Palatal	75	44 (53.7)	31 (44.9)		

Position of the Canalis sinuosus (CS):

1 – Palatal to central incisor, 2 – Palatal to lateral incisor, 3 – Palatal to canine, 4 – Distopalatal to central incisor, 5 – Distopalatal to lateral incisor, 6 – Distopalatal to canine, 7 – Mesiopalatal to central incisor, 8 – Mesiopalatal to lateral incisor, 9 – Mesiopalatal to canine.

In general, males exhibited larger measurements in both diameter and distance from various CS landmarks compared to females. Notably, the diameter on the left side was significantly larger in males (0.8 ± 0.29) than in females (0.7 ± 0.23), with a p-value of 0.008. Although the right side showed a similar pattern, the difference was not statistically significant. The distance from the CS to the alveolar crest was similar for both genders. However, the distance from the CS to the buccal cortical plate was significantly greater in males, measuring 7.78 mm on the left and 7.53 mm on the right, with a p-value of less than 0.001. Furthermore, the distance from the CS to the floor of the nasal cavity was significantly greater on the right side, with males having a mean value of 12.44 mm compared to 10.89 mm in females, and a p-value of 0.011.

**Figure 5.**
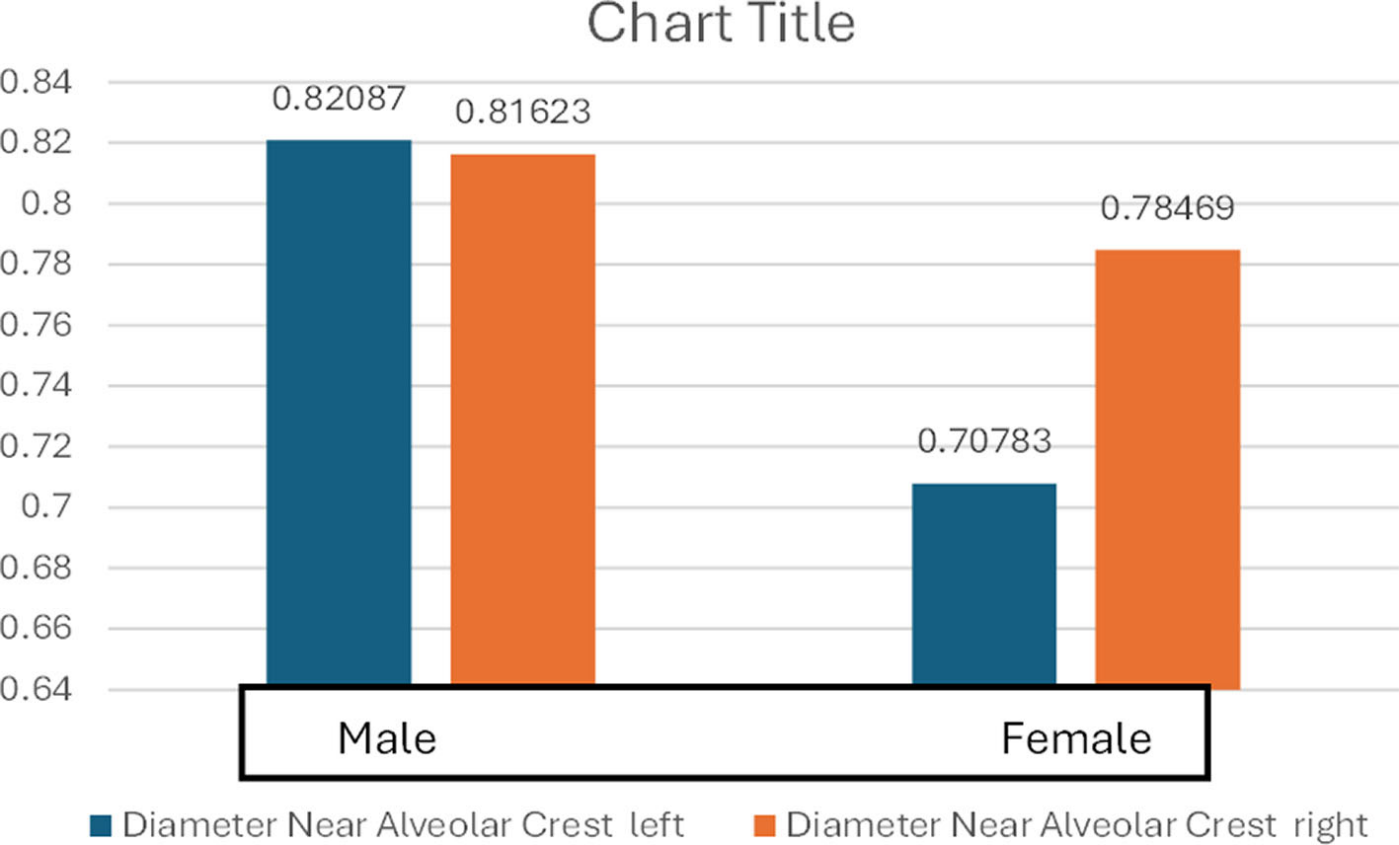
Diameter of the CS near the alveolar crest in males and females.

**Figure 6.**
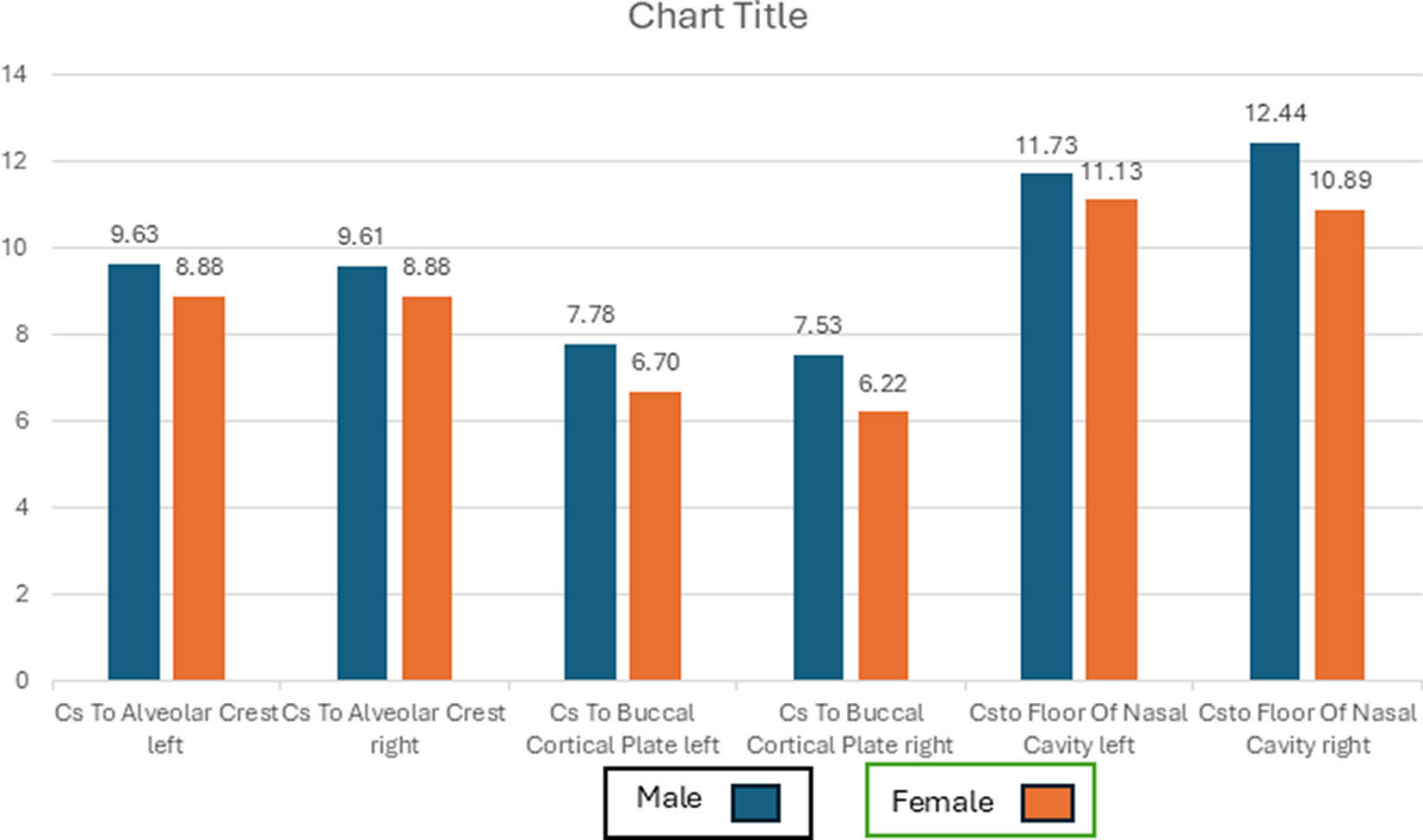
Distance of the CS on both sides from the alveolar crest, buccal cortical plate, and nasal floor in males and females.

Overall, notable differences were found in the diameter near the alveolar crest (left), the distance from the CS to the buccal cortical plate (both sides), and the distance from the CS to the floor of the nasal cavity (right), all of which were greater in males. Although these sex-related differences are modest in absolute magnitude, they suggest that a uniform safety margin may not be appropriate for all patients, particularly in anterior maxillary regions with limited bone volume. Other parameters did not exhibit statistically significant differences between the two groups ( Figures 5, 6).

## Discussion

This retrospective cone-beam CT study of 245 Indian adults demonstrated that the canalis sinuosus was detectable in approximately two-thirds of scans, most commonly as a bilateral structure coursing palatally in the lateral incisor region. The canal diameter near the alveolar crest was slightly below 1 mm, and measurable distances from the CS to the alveolar crest, buccal cortical plate, and nasal floor were on the order of several millimeters, indicating a relatively narrow safety zone for anterior maxillary procedures. In addition, males generally exhibited larger canal diameters and greater distances to the buccal cortical plate and nasal floor than females, highlighting sex-related morphometric variation that may be relevant for implant planning.

Compared with previous CBCT studies of the CS conducted primarily in non-Indian populations, the present work provides one of the few detailed descriptions of CS prevalence, tooth-related location, and morphometry in an Indian adult cohort. By additionally quantifying sex-related differences in linear distances to surgically relevant landmarks, this study extends available normative data and helps refine population-specific safety margins for implant placement and other anterior maxillary interventions.

Canalis Sinuosus (CS) is an anatomical variation of the ASAN, responsible for carrying the neurovascular bundle that supplies blood and innervates the incisors, canines, and adjacent soft tissues. ^8^ Dental implants are the preferred treatment for replacing one or more missing teeth in the anterior maxilla, a region noted for its high vascularity and trabecular density. ^9^ Understanding the anatomical structures in this area is crucial for achieving predictable and safe surgical outcomes. Due to the limitations of 2D images, such as structural superimpositions in the anterior region, CBCT offers precise measurements of surface distances and accurately associates teeth with vital structures. These advantages have made CBCT an indispensable imaging tool in dentistry. ^10^ CS is recognized as a distinct anatomical structure, with its prevalence reported to range from 66.5% to 100% across various population groups. The present study found that 67.3% of the patients presented with CS, consistent with the results of Orhan K et al., Ghandourah AO et al., Anatoly A et al., and Aoki R et al. ^11– 14^ When detected, CS is usually found bilaterally, with its presence documented to range from 46% to 100%. ^4, 12– 14^ In our study, 55.9% of the subjects exhibited CS bilaterally, consistent with the findings of Aoki et al. ^14^ CS was absent in 32.7% of patients, and 11.4% had it unilaterally. ^15^ The identification of bilateral CS (CS) can be influenced by multiple variables, such as the choice of imaging modality, voxel size, criteria for study inclusion and exclusion, the observer’s expertise, and the presence or absence of imaging artifacts. ^16^ When each side was assessed individually, no significant findings were observed regarding the sides. These results align with the observations documented by Wanzeler AM et al. ^17^ and Gurler G. et al. ^18^ However, Manhães et al. noted a higher occurrence of CS on the left side in their study. ^2^

### Position of CS

While CS can present in various anatomical locations in the anterior maxilla, it predominantly occurs in the region adjacent to the palate in the incisor and canine areas. ^19^ This distribution aligns with the findings of our study and corroborates the observations documented by Manhães Júnior LR et al. ^2^ as well as Anatoly A et al. ^13^ and Samunahmetoglu E and Kurt MH. ^5^

### Metric evaluation

**Diameter:** The mean diameter of the CS is generally reported to be around 1 mm. ^14, 19, 20^ However, in our study, we observed a mean diameter slightly less than 1 mm, which is consistent with the findings of Ghandourah AO et al. ^12^ and Khojastepour L and Akbarizadeh F, ^15^ although other studies have documented measurements exceeding 1 mm. ^4, 5, 21, 22^ While the precise relationship between canal diameter and the likelihood of complications remains uncertain, it is noteworthy that a larger neurovascular bundle may increase the risk of surgical issues, particularly bleeding. Furthermore, an increased canal diameter can potentially lead to misinterpretation of periapical lesions on standard radiographs. ^5^

**Distance from Adjacent Structures:** In our study, the distance measured from the CS to the alveolar crest was slightly over 9 mm, closely aligning with the findings of Manhães et al. ^2^ In contrast, Gürler et al. reported a significantly greater distance of 16.81 mm. ^18^ Meanwhile, Samunahmetoğlu E. and Kurt MH ^5^ found a distance of 7.71 mm, and Beyzade Z et al. ^16^ reported 5.87 mm, both slightly less than our result. The distance from the CS to the buccal cortical plate was over 7 mm, consistent with the findings of Manhães et al. ^2^ However, Beyzade Z et al. ^16^ and Samunahmetoğlu, E. and Kurt, M.H. ^5^ found it to be less than 5 mm in their study. In our study, the distance measured between the CS and the nasal cavity exceeded 11.4 mm, aligning with the observations reported by Manhães et al. ^2^

### Occurrence

The presence or absence of CS, as well as its lateral positioning, did not demonstrate a statistically significant association with sex. In both male and female subjects, the proximity of CS was closest to the lateral incisor, followed by the lateral incisor and the canine on both sides, which was statistically insignificant. The location of CS relative to the tooth also exhibited no significant differences based on sex, with it most frequently positioned palatally, followed by disto-palatal and then mesio-palatal. In the analysis of CS in relation to sex, no statistically significant correlations were identified concerning its presence, position, proximity to teeth, or location. These findings are consistent with the results reported by Aoki R et al. ^14^

### Gender-based evaluation

The CS exhibited a larger diameter near the alveolar crest in males compared to females. This difference was statistically significant on the left side, while no significant variation was observed on the right side. Gurler et al., Machado et al., and Shan et al. found that the diameter was greater in males. ^18, 20, 23^ Gurler et al. suggested that the increased diameter in males could be attributed to generally wider anatomical structures. ^18^ However, this observation contrasts with the findings of Samunahmetoglu, E and Kurt, M.H., and Von Arx et al., who reported no correlation between diameter and sex. ^5, 22^ Our study revealed that the distance from the CS to the alveolar crest was greater in males than in females on both sides, though this difference was not statistically significant. However, the distance of CS to the buccal cortical plate was significantly larger in males on both sides and was statistically significant. Despite the overall trend of larger measurements in males, a study by Manhães reported a statistically significant difference in the distance from the CS to the buccal cortical plate in females. ^2^ Furthermore, the distance from the CS to the floor of the nasal cavity on the left side was slightly greater in males than in females on both sides. However, this difference did not reach statistical significance. These findings are consistent with the observations made by Manhães et al. and Samunahmetoglu, E., and Kurt, M.H., who also reported that all measured parameters tended to be greater in males, in line with our study results. ^2, 5^

Overall, significant differences were observed in the diameter near the alveolar crest (left), distance from the CS to the buccal cortical plate (both sides), and distance from the CS to the floor of the nasal cavity (right), all of which were larger in males. Between the two groups, no statistically significant differences were observed.

### Limitations and future directions

This study has several limitations. First, its retrospective, single-centre design and purposive sampling may limit the generalizability of the findings to other Indian or South Asian populations. Second, the analysis relied solely on CBCT imaging and was not correlated with clinical outcomes, such as the occurrence of CS-related complications after implant placement. Third, only patients with intact maxillary incisors and canines were included, which may introduce selection bias. Future multi-centre studies that combine CBCT evaluation with clinical follow-up could help validate and further refine the morphometric parameters reported here.

## Conclusion

In this retrospective cone-beam CT study of 245 Indian adults, the canalis sinuosus was detected in approximately two-thirds of scans, most often as a bilateral structure coursing palatally in the lateral incisor region. The canal lay at measurable distances from the alveolar crest, buccal cortical plate, and nasal floor, and males tended to exhibit larger canal diameters and greater linear distances than females. These population- and sex-specific morphometric data underscore the importance of careful preoperative CBCT assessment of the anterior maxilla and may assist clinicians in defining patient-specific safety margins for implant placement and other surgical interventions, thereby reducing the risk of CS-related neurovascular complications.

## Ethics and consent

The study was reviewed and approved by the Manipal College of Dental Sciences Institutional Ethics Committee with approval number (protocol number 24150/2025) dated 21/1/2025. All procedures performed involving human participants were in accordance with the ethical standards of the institutional committee and with the 1964 Declaration of Helsinki and its later amendments. Written informed consent was obtained from all participants for their involvement in the study.

## Author contributions

Ceena Denny – Conceptualization, Investigation, Formal analysis Writing original draft, Design of work, review & editing, Methodology

Srikant Natarajan – Formal analysis, Methodology, Design of work

Hannah Haneef – Data curation, Validation, Investigation

Shubha B S – Investigation, Resources

Divyam Sen – Investigation, Resources

Aishwarya Sukumaran Nair – Conceptualization, Formal analysis, Final approval

## Data Availability

Figshare: Cone-Beam CT Assessment of the Canalis Sinuosus in an Indian Population: A Retrospective Imaging Study. https://doi.org/10.6084/m9.figshare.30998170.v2 ^24^ This project contains the following underlying data:
• CS.xlsx This project contains the following extended data:
• Tables.pdf Data are available under the terms of the Creative Commons Attribution 4.0 International license (CC-BY 4.0).
