# Supplementary material for: Cone-Beam CT Assessment of the Canalis Sinuosus in an Indian Population: A Retrospective Imaging Study
Source: F1000Res. 2026 Jul 16;15:174. Originally published 2026 Feb 3. [Version 4] doi: 10.12688/f1000research.176689.4 (PMC13270192; doi:10.12688/f1000research.176689.4)
Supplement: Peer review history

# Reviewer report: Reviewer response for version 4

**DOI:** 10.5256/f1000research.204012.r502048

**Published:** 2026-07-20

**Authors:** SEMA KAYA

**Affiliations:** 1 Van Yüzüncü Yıl Üniversitesi, Van, Turkey

The revised version of the manuscript has addressed the concerns raised in my previous review. The authors have clarified the imaging parameters by specifying the exact field of view and voxel size, expanded the limitations section, improved the discussion regarding the clinical interpretation of statistically significant findings, and appropriately acknowledged the limitations associated with applying measurements obtained from dentate individuals to implant planning in edentulous patients. The manuscript also now recognizes the increased risk of Type I error due to multiple comparisons and justifies the exploratory nature of the analyses. Furthermore, the low reliability of canal diameter measurements has been discussed appropriately, with a clear statement that these measurements should not be used in isolation for clinical decision-making. The quality and interpretability of the figures have also been improved by correcting the graphs and adding arrows to identify the Canalis Sinuosus in the representative images.

 Overall, the revisions have substantially strengthened the manuscript, and I believe that the concerns raised in my previous report have been satisfactorily addressed. The manuscript is now suitable for approval.

Is the work clearly and accurately presented and does it cite the current literature?

Yes

If applicable, is the statistical analysis and its interpretation appropriate?

Yes

Are all the source data underlying the results available to ensure full reproducibility?

Partly

Is the study design appropriate and is the work technically sound?

Yes

Are the conclusions drawn adequately supported by the results?

Yes

Are sufficient details of methods and analysis provided to allow replication by others?

Yes

Reviewer Expertise:

My Academic Interests: Oral and Maxillofacial Radiology, Cone-Beam Computed Tomography (CBCT) applications and interpretation, Maxillofacial anatomical variations and clinical significance, Radiological assessment for dental implant planning and risk analysis, Temporomandibular joint (TMJ) imaging, Artificial intelligence applications in radiology, Health Law and medical malpractice, Legal responsibilities of physicians and patient rights

I confirm that I have read this submission and believe that I have an appropriate level of expertise to confirm that it is of an acceptable scientific standard.

# Reviewer report: Reviewer response for version 3

**DOI:** 10.5256/f1000research.200361.r488775

**Published:** 2026-06-15

**Authors:** SEMA KAYA

**Affiliations:** 1 Van Yüzüncü Yıl Üniversitesi, Van, Turkey

This CBCT-based study evaluating the Canalis Sinuosus in an Indian population provides important anatomical data that may inform implant planning in the anterior maxilla. The calculation of sample size through power analysis, the statistical assessment of inter-observer and intra-observer agreement, and the consistency of findings with the existing literature are notable strengths of this work. However, several issues need to be addressed before the manuscript can be considered for indexing.

 Expressions such as "medium FOV" and "voxel size <200 µm" are subjective and insufficient. The exact FOV dimensions (e.g., 8×8 cm or 10×10 cm) and the precise voxel size (e.g., 150 µm, 180 µm, or 200 µm) must be clearly stated.

 In Table 3, the intraclass correlation coefficient (ICC) for canal diameter near the alveolar crest is reported as 0.353 (p = 0.089). This value indicates poor reliability and is not statistically significant. This finding should be acknowledged as a limitation in the discussion section, and it should be emphasized that canal diameter measurements should not be used in isolation for clinical decision-making.

 Numerous statistical tests have been performed across multiple parameters, increasing the risk of Type I error. Either a correction for multiple comparisons (e.g., Bonferroni) should be applied, or the justification for not applying such a correction (e.g., "this is an exploratory study") should be explicitly stated.

 Although the study was conducted exclusively in dentate individuals (with intact maxillary incisors and canines), the findings are repeatedly presented as safety margins for implant planning. Following tooth extraction, alveolar ridge undergoes dimensional changes in height, width, and bone density. Therefore, distance measurements obtained from dentate individuals cannot be directly applied to edentulous sites. This limitation should be emphasized not only in the limitations section but also prominently in the introduction of the discussion.

 The canal diameter on the left side was found to be statistically significantly larger in males than in females (0.8 mm vs. 0.7 mm; p = 0.008). However, the absolute difference is only 0.1 mm. Similarly, differences in buccal cortical distances are on the order of a few millimeters. It should be clearly stated that statistical significance does not imply clinical significance, and that such small absolute differences should not be used as sole determinants in clinical decision-making.

 In Figure 5, the data for males and females are presented in a graph type that is not suitable for direct comparison. The figure should be converted to a grouped bar chart format. The legend should use "Male" and "Female" instead of "M" and "F".

 The exact location of the Canalis Sinuosus is not clearly discernible in Figures 1–4 due to the absence of anatomical indicators. Each figure should include an arrow or arrowhead clearly pointing to the CS. Adjustments to image contrast and brightness may also be considered to enhance visibility.

Is the work clearly and accurately presented and does it cite the current literature?

Yes

If applicable, is the statistical analysis and its interpretation appropriate?

Yes

Are all the source data underlying the results available to ensure full reproducibility?

Partly

Is the study design appropriate and is the work technically sound?

Yes

Are the conclusions drawn adequately supported by the results?

Yes

Are sufficient details of methods and analysis provided to allow replication by others?

Yes

Reviewer Expertise:

My Academic Interests:Oral and Maxillofacial RadiologyCone-Beam Computed Tomography (CBCT) applications and interpretationMaxillofacial anatomical variations and clinical significanceRadiological assessment for dental implant planning and risk analysisTemporomandibular joint (TMJ) imagingArtificial intelligence applications in radiologyHealth Law and medical malpracticeLegal responsibilities of physicians and patient rights

I confirm that I have read this submission and believe that I have an appropriate level of expertise to confirm that it is of an acceptable scientific standard, however I have significant reservations, as outlined above.

# Reviewer report: Reviewer response for version 2

**DOI:** 10.5256/f1000research.196924.r468972

**Published:** 2026-04-15

**Authors:** Prashanti Eachempati

**Affiliations:** 1 Peninsula Dental School, University of Plymouth, Plymouth, Devon, UK

The manuscript addresses a clinically relevant anatomical structure and provides useful CBCT-based data in an Indian population, which is currently underrepresented in the literature. The study is generally well conducted, clearly presented, and has strong potential. However, a few key aspects should be addressed to strengthen its scientific rigour, clarity, and clinical interpretation.

 At the outset, I would like to highlight a potential issue in the **Results section regarding the units of measurement. Some distances appear to be reported in centimetres rather than millimetres.** I think this is a typographical error; however, if so, this represents a major issue and should be carefully reviewed and corrected throughout the manuscript to ensure accuracy and consistency.

 First, the interpretation of the statistical findings would benefit from going beyond p-values alone. **While several differences are reported as statistically significant, their clinical relevance is not always clear.** For example, the reported difference in canal diameter between males and females on the left side (0.8 mm vs 0.7 mm, p = 0.008) is statistically significant but small in absolute magnitude (~0.1 mm). It would be helpful for the authors to clarify whether such a difference is likely to be clinically meaningful in practice. Similarly, differences in distances to anatomical landmarks (e.g., buccal cortical plate, nasal floor) should be interpreted in terms of their practical significance, rather than statistical significance alone.

 Second, given that **multiple statistical comparisons have been performed across several outcomes and subgroups, there is an increased likelihood of type I error** (i.e., identifying statistically significant findings by chance). While this is understandable in an exploratory anatomical study and may not require formal adjustment, it would be appropriate for the authors to acknowledge this as a limitation and interpret statistically significant findings with some caution.

 In addition, a few methodological clarifications would improve transparency and reproducibility. The **measurement protocol should be described in a clearer,** step-by-step manner, including how anatomical landmarks were defined and how measurements were obtained on CBCT images. While **inter-observer agreement** for detection is reported, reliability **for the morphometric measurements** themselves is not described and should be clarified. It would also be helpful to **specify whether measurements were taken once or repeated and averaged.**

 One important point relates to the **study population and its clinical applicability.** The inclusion criteria specify patients with intact maxillary incisors and canines (i.e., dentulous individuals), whereas the discussion and conclusions extend the implications to implant planning, which commonly involves partially or fully edentulous patients. As alveolar bone morphology changes following tooth loss, the spatial relationships and measurements reported here may not directly translate to edentulous sites. The authors may wish to acknowledge this as a limitation and clarify the extent to which these findings can be applied to implant planning scenarios.

 Related to this, while the **anatomical findings are valuable, their direct translation to implant planning should be interpreted with caution.** The clinical significance of this study lies primarily in enhancing anatomical awareness and highlighting the presence and typical location of the canalis sinuosus, rather than defining precise surgical safety margins. The manuscript would benefit from reframing this distinction more explicitly.

 The conclusions are generally consistent with the results; however, they could be slightly tempered in terms of clinical implications. **The strength of the conclusions should reflect the observational and imaging-based nature of the study.**

 Finally, minor language editing would help reduce some repetition and improve clarity in the discussion.

 Overall, this is a well-conducted and clinically relevant study, and with the above clarifications and refinements, particularly around measurement reporting, clinical interpretation, and applicability, it would be a stronger paper.

Is the work clearly and accurately presented and does it cite the current literature?

Yes

If applicable, is the statistical analysis and its interpretation appropriate?

Partly

Are all the source data underlying the results available to ensure full reproducibility?

Yes

Is the study design appropriate and is the work technically sound?

Yes

Are the conclusions drawn adequately supported by the results?

Partly

Are sufficient details of methods and analysis provided to allow replication by others?

Partly

Reviewer Expertise:

Prosthodontics and Implant Dentistry; Evidence-Based Dentistry; Evidence Synthesis (Systematic Reviews and Meta-analysis); GRADE Methodology and Certainty of Evidence Assessment; Clinical Epidemiology; Health Research Methods

I confirm that I have read this submission and believe that I have an appropriate level of expertise to confirm that it is of an acceptable scientific standard, however I have significant reservations, as outlined above.

# Response

**Published:** 2026-04-25

**Authors:** CEENA DENNY

**Affiliations:** Manipal College of Dental Sciences,Mangalore,Manipal Academy of Higher Education,Manipal-575001, India

Comment 1. I would like to highlight a potential issue in the **Results section regarding the units of measurement. Some distances appear to be reported in centimetres rather than millimetres.** I think this is a typographical error; however, if so, this represents a major issue and should be carefully reviewed and corrected throughout the manuscript to ensure accuracy and consistency.

**Response: Made the changes in the manuscript in page 3**

 Comment 2. The interpretation of the statistical findings would benefit from going beyond p-values alone. **While several differences are reported as statistically significant, their clinical relevance is not always clear.** For example, the reported difference in canal diameter between males and females on the left side (0.8 mm vs 0.7 mm, p = 0.008) is statistically significant but small in absolute magnitude (~0.1 mm). It would be helpful for the authors to clarify whether such a difference is likely to be clinically meaningful in practice. Similarly, differences in distances to anatomical landmarks (e.g., buccal cortical plate, nasal floor) should be interpreted in terms of their practical significance, rather than statistical significance alone.

**Response: Made the changes in the discussion and also added about the practical significance (PAGE 6-13)**

 Comment 3. Second, given that multiple statistical comparisons have been performed across several outcomes and subgroups, there is an increased likelihood of type I error (i.e., identifying statistically significant findings by chance). While this is understandable in an exploratory anatomical study and may not require formal adjustment, it would be appropriate for the authors to acknowledge this as a limitation and interpret statistically significant findings with some caution

**Response: Added in Page 13**

 Comment 4. In addition, a few methodological clarifications would improve transparency and reproducibility. The measurement protocol should be described in a clearer, step-by-step manner, including how anatomical landmarks were defined and how measurements were obtained on CBCT images.

**Response: Adding as a supplemental file**

 Comment **5. **While inter-observer agreement for detection is reported, reliability for the morphometric measurements themselves is not described and should be clarified. It would also be helpful to specify whether measurements were taken once or repeated and averaged

**Response: Added as table 3**

 We have now clarified the reliability of the morphometric measurements in the revised manuscript. A subset of scans (n = 40) was re-evaluated, and all measurements were performed twice by the same observer at different time points to minimize bias. The final values used in the analysis were obtained by averaging the two measurements.

 In addition, intra-observer reliability was assessed using intraclass correlation coefficients (ICC). The results have been included in the manuscript, demonstrating excellent reliability for linear measurements (ICC ranging from 0.956 to 0.984; p < 0.001) and lower reliability for diameter near the alveolar crest (ICC = 0.353; p = 0.089). This clarification has been incorporated into both the Methods and Results sections of the revised manuscript.

 Comment 6. One important point relates to the study population and its clinical applicability. The inclusion criteria specify patients with intact maxillary incisors and canines (i.e., dentulous individuals), whereas the discussion and conclusions extend the implications to implant planning, which commonly involves partially or fully edentulous patients. As alveolar bone morphology changes following tooth loss, the spatial relationships and measurements reported here may not directly translate to edentulous sites. The authors may wish to acknowledge this as a limitation and clarify the extent to which these findings can be applied to implant planning scenarios.

 Related to this, while the anatomical findings are valuable, their direct translation to implant planning should be interpreted with caution. The clinical significance of this study lies primarily in enhancing anatomical awareness and highlighting the presence and typical location of the canalis sinuosus, rather than defining precise surgical safety margins. The manuscript would benefit from reframing this distinction more explicitly.

**Response: Addressed under limitations and futuristic directions Page-13**

 Comment 7. The conclusions are generally consistent with the results; however, they could be slightly tempered in terms of clinical implications. The strength of the conclusions should reflect the observational and imaging-based nature of the study.

**Response: Addressed in Page 14**

# Reviewer report: Reviewer response for version 2

**DOI:** 10.5256/f1000research.196924.r468661

**Published:** 2026-04-10

**Authors:** P Kalyana Chakravarthy

**Affiliations:** 1 Manipal College of Dental Sciences, Manipal, Karnataka, India

The authors have made the necessary changes.  APPROVED.

Is the work clearly and accurately presented and does it cite the current literature?

Yes

If applicable, is the statistical analysis and its interpretation appropriate?

Partly

Are all the source data underlying the results available to ensure full reproducibility?

Yes

Is the study design appropriate and is the work technically sound?

Yes

Are the conclusions drawn adequately supported by the results?

Yes

Are sufficient details of methods and analysis provided to allow replication by others?

Partly

Reviewer Expertise:

Oral Epidemiology

I confirm that I have read this submission and believe that I have an appropriate level of expertise to confirm that it is of an acceptable scientific standard.

# Reviewer report: Reviewer response for version 1

**DOI:** 10.5256/f1000research.194778.r455630

**Published:** 2026-02-11

**Authors:** P Kalyana Chakravarthy

**Affiliations:** 1 Manipal College of Dental Sciences, Manipal, Karnataka, India

The authors have done commendable work. However, there is scope for improvement. Below are my suggestions to improve the manuscript

- Please report the parameters used in the sample size calculation, specifically zα/2, p, and e.
- Specify the duration and time period of data collection.
- Clearly state the study design.
- Indicate the total number of CBCT images screened, the number included for analysis, and provide reasons for exclusion with corresponding numbers.
- The results section states that Pearson’s Chi-square test showed a significant *correlation* between CS location and tooth proximity on the left (χ² = 18.164, df = 4, p = 0.001) and right sides (χ² = 9.521, df = 4, p = 0.049).

- Please present these results in tabular format.
- Additionally, the term “correlation” is inappropriate for Chi-square analysis and should be replaced with “association.”

- Figure 5 requires improvement: Convert it to a two-dimensional graph. Display male and female bars adjacent to each other. Round all numerical values to two decimal places for clarity. Replace M and F with Male and Female in the legend.
- In Table 1, the left and right position categories (0–9) should be clearly described.
- Replace M/F with Male/Female throughout the table

- Cohen’s K is mentioned in the abstract. Please elaborate on this in the manuscript: Add details in the Methodology regarding the number of examiners and the corresponding Cohen’s kappa coefficient. For metric measurements, please report the Intraclass Correlation Coefficient (ICC).
- Specify the normality test used prior to applying the Student’s *t*-test in the Statistical Analysis section.
- The following statement could not be found in Table 1:
- “Furthermore, the distance from the CS to the floor of the nasal cavity was significantly greater on the right side, with males having a mean value of 12.44 mm compared to 10.89 mm in females, and a p-value of 0.011 (Table 1).”
- In the Discussion, the sentence referring to Samunahmetoglu, Kurt, and Von Arx should follow a uniform author citation style, for example: Samunahmetoglu and Kurt, and Von et al., with appropriate references cited immediately after the author names.
- In several instances (e.g., Manhães, Von Arx, Gurler), authors are mentioned without using “et al.” where applicable. Please revise for consistency.
- The manuscript shows inconsistent citation formatting, with some instances listing full author names and others using only et al.. For few citations in text, authors with last names were written, and at few instances, only the last name et al was written. A uniform citation pattern should be followed throughout.
- To avoid ambiguity, it is recommended that citations be placed immediately after author names. For ex: The present study found that 67.3% of the patients presented with CS, consistent with the results of Ghandourah AO et al., Orhan K et al., Anatoly A et al., and Aoki R et al. ^11–14.^ Ghandourah AO et al., in the reference list was 12 and Orhan K et al was 11.
- Where a range of prevalence is reported (e.g., 46%–100%), multiple references should be cited. Currently, only a single reference is provided.

Is the work clearly and accurately presented and does it cite the current literature?

Yes

If applicable, is the statistical analysis and its interpretation appropriate?

Partly

Are all the source data underlying the results available to ensure full reproducibility?

Yes

Is the study design appropriate and is the work technically sound?

Yes

Are the conclusions drawn adequately supported by the results?

Yes

Are sufficient details of methods and analysis provided to allow replication by others?

Partly

Reviewer Expertise:

Oral Epidemiology

I confirm that I have read this submission and believe that I have an appropriate level of expertise to confirm that it is of an acceptable scientific standard, however I have significant reservations, as outlined above.
